# Evaluation of salivary tumor necrosis factor *α* as a diagnostic biomarker in oral submucosal fibrosis and squamous cell carcinoma of the oral cavity and oropharynx: a cross sectional observational study

**DOI:** 10.3389/froh.2024.1375162

**Published:** 2024-03-26

**Authors:** Sabiha Abdul Aziz Shaikh, Ceena Denny E, Reshma Kumarchandra, Srikant Natarajan, Johan Sunny, Nandita Shenoy, Nandita K. P

**Affiliations:** ^1^ Department of Oral Medicine and Radiology, Manipal College of Dental Sciences, Mangalore, Manipal Academy of Higher Education, Manipal, India; ^2^ Department of Biochemistry, Kasturba Medical College, Mangalore, Manipal Academy of Higher Education, Manipal, India; ^3^ Department of Oral and Maxillofacial Pathology, Manipal College of Dental Sciences, Mangalore, Manipal Academy of Higher Education, Manipal, India; ^4^ Department of Radiation Oncology, Kasturba Medical College, Mangalore, Manipal Academy of Higher Education, Manipal, India

**Keywords:** biomarkers, oral submucous fibrosis, TNF-α, squamous cell carcinoma, oral cavity, oropharynx

## Abstract

**Introduction:**

Tumor necrosis factor *α* (TNF-α) is known to be associated with chronic inflammation, and its expression has been shown to increase in advanced cancers. Chronic inflammation is a characteristic feature of oral submucous fibrosis (OSMF), which is a potentially malignant disorder (PMD). Squamous cell carcinoma (SCC) is associated with considerable mortality and morbidity and an early detection or monitoring would greatly help in achieving an effective cure. TNF-α was thus evaluated for use as a biomarker in the present study according to the stage of OSMF and histological grade of SCC in the oral cavity and oropharynx.

**Methods:**

This study included 45 patients divided into 3 groups—OSMF group, SCC group and control group—each comprising 15 participants. Saliva samples were collected from each patient, and salivary TNF-α levels were estimated using an ELISA kit.

**Results:**

Statistical analysis revealed no significant differences in TNF-α levels among the OSMF, SCC and control groups; however, there was an increase in the salivary TNF-α level in patients with stage 3 disease according to the clinical stage of OSMF, for which the *p* value was 0.027.

**Discussion:**

An increase in the TNF-α concentration with increasing clinical stage suggested a role for TNF-α in the spread of OSMF involvement in anatomical structures of the oral cavity and oropharynx. No significant difference in salivary TNF-α levels was noted among the OSMF, SCC and control groups.

**Conclusion:**

The study showed a positive correlation of TNF-α with increasing stages of OSMF but was not a reliable biomarker in the categorization of the same.

## 1 Introduction

The WHO working group in 2020 defined Oral Potentially Malignant Disorders (OPMD) as “any oral mucosal abnormality that is associated with a statistically increased risk of developing oral cancer” (1). OSMF is a debilitating oral disorder characterized by progressive fibrosis of the submucosal tissues in the mouth. This condition primarily affects individuals residing in Southeast Asia, where it has reached alarming levels. The age range of those affected is vast, spanning from young children to elderly people. One of the most concerning aspects of OSMF is its potential to transform into a malignancy. Studies suggest that the malignant transformation rate of OSMF varies between 1.5% and 15%, highlighting the severity of this condition (2). According to estimates, the prevalence of OSMF in India ranges from 0.2% to 2.3% in men and 1.4% to 4.6% in women, with a wide age range of 11–60 years (3). In 1952, Schwartz first identified oral submucosal fibrosis (OSMF) as “Atropica idiopathica mucosae oris,” and Jens J. Pindborg subsequently described it as “an insidious, chronic disease that affects any part of the oral cavity and sometimes the pharynx. Although occasionally preceded by, or associated with, the formation of vesicles, it is always associated with a juxtaepithelial inflammatory reaction followed by fibroelastic change in the lamina propria and epithelial atrophy that leads to stiffness of the oral mucosa and causes trismus and an inability to eat” (4). Compared to other OPMDs, individuals with OSMF have a higher tendency of developing SCC according to studies, the rate of malignant transformation was seen between 1.9% and 9%. According to a study done by Guo et al. in China in 2011, oral cancer arising from OSMF is clinically more aggressive and has higher rates of recurrence and metastasis than standard OSCC (5).

The prevalence of oral cancer is significant, as it impacts various parts of the body, such as the lips and oral cavity including the tongue, upper and lower gums, floor of the mouth, palate and other areas of the mouth. The World Health Organization (WHO) has highlighted the South East Asia region with a notably increased occurrence of oral cancers (6). This can be attributed to the widespread use of areca nuts, alcohol, poor nutritional status, and tobacco, particularly smokeless tobacco (7). Oral cancer is currently the sixth most common type of cancer worldwide. In particular, India has the highest incidence rate, accounting for one-third of the global burden (8). In India, squamous cell carcinomas (SCCs) originating from the lining of the mucous membrane are responsible for 90%–95% of all new cases of OC (9).

Biomarkers are biological indicators that are becoming increasingly valuable in the diagnosis, grading, monitoring, and prognosis of various conditions. Moreover, they play a crucial role in developing specialized therapeutic approaches (10). Salivary biomarkers have emerged as promising avenues for diagnosing and monitoring OSMF and OSCC. By analysing specific substances present in saliva, healthcare professionals can gain valuable insights into the progression and severity of these conditions. Moreover, the noninvasive nature of saliva collection makes it a preferred choice for patients, eliminating the need for invasive procedures (2). Currently, patient management relies on traditional histologic parameters such as TNM and tumor grading. However, there is ongoing research into new salivary biomarkers that exhibit high sensitivity and specificity in detecting oral cancer in saliva (11). The aim is to improve patient treatment and survival by characterizing the diversity of cancer and stratifying patients according to specific treatments (12). In the case of patients with OSMF, the upregulation of salivary biomarkers can be utilized to improve evaluation, identify transformation to malignancy, and facilitate targeted therapies (1). Hence the present study was done to investigate if TNF α can be used as a biomarker in the assessment of various stages of OSMF and histological grades of squamous cell carcinoma.

Tumor necrosis factor alpha (TNF-α) is a pivotal mediator of inflammation and possesses the unique capability to both induce and repair tissue damage. Additionally, it facilitates the elimination of malfunctioning cells at the site of inflammation while promoting the growth of fibroblasts (13). Clinical research has shown that patients with OSMF exhibit abnormal expression of TNF-α and other growth factors (14). Arecoline, a compound derived from the areca nut, is believed to promote the production of TNF-α in oral tissues, thereby triggering cellular inflammation (2). Currently, the management of OSMF remains primarily focused on alleviating symptoms, as standardized treatment guidelines have not yet been established. Consequently, it is imperative to explore and utilize molecular biomarkers in this context (2). Numerous studies have indicated that patients diagnosed with OSMF exhibit significantly elevated levels of plasma TNF-α. Consequently, TNF inhibitor therapies have emerged as potential treatments for OSMF, given the observed correlation between the condition and heightened TNF-α levels (14). Therefore, the primary objective of this study was to investigate the potential utility of TNF-α as a biomarker for assessing different stages of OSMF and histological grades of squamous cell carcinoma (SCC).

## 2 Materials and methods

### 2.1 Study subjects and the source of the data

The participants in this study were patients who sought treatment at the Department of Radiation Oncology, Kasturba Medical College Hospital, and the Department of Oral Medicine and Radiology, Manipal College of Dental Sciences in Mangalore. Prior to conducting the study, approval was obtained from the Institutional Ethics Committee at the Manipal College of Dental Sciences, Mangalore, with the protocol reference number 20094. Additionally, informed consent was obtained from each participant.

### 2.2 Inclusion and exclusion criteria

#### 2.2.1 Inclusion criteria

The study consisted of 45 participants, ranging in age from 20 to 65 years, who were divided into three groups. Group 1 included 15 patients with OSMF who were diagnosed and graded using the functional and clinical grading system developed by Haider et al. (15). Group 2 consisted of 15 patients with SCC who were histopathologically diagnosed using Broder's grading system (16). Group 3 consisted of healthy individuals of similar age who served as matched controls. In Group 1, individuals were diagnosed based on clinical examination, while individuals in Groups 1 and 2 had not received or were not currently receiving any definitive therapy, such as radiotherapy, chemotherapy, surgery, or any other treatment for SCC.

#### 2.2.2 Exclusion criteria

1. To ensure the accuracy of our findings, we excluded individuals who had conditions known to increase cytokine levels in saliva, such as rheumatoid arthritis, osteoporosis, or chronic obstructive pulmonary disease.
2. Additionally, we excluded participants who had a history of taking medications for any other medical condition or were using drugs such as antihistamines, antidepressants, antihypertensives, anticholinergics, or bronchodilators.

### 2.3 Study population size

Based on the parameter of SCC analysed by Deepthi et al. (17) the standard deviation for group I was 56.05, the standard deviation for group II was 20.94.

The main difference of values that was considered to be clinically relevant was 40 units. Using the sample size calculation formula for two means,

$$\begin{array}{rl} & \mathrm{With}\;Z\;\mathrm{value}\;\mathrm{for}\;\alpha \;1.959\;\mathrm{and}\;Z\;\mathrm{value}\;\mathrm{for}\;\beta \;\mathrm{was}\;0.84,\;\mathrm{we}\;\mathrm{arrived}\\ & \;\mathrm{at}\;a\;\mathrm{sample}\;\mathrm{size}\;\mathrm{of}\;15\;\mathrm{for}\;\mathrm{each}\;\mathrm{group}.\;\mathrm{The}\;\mathrm{patients}\;\mathrm{were}\\ & \;\mathrm{selected}\;\mathrm{as}\;\mathrm{per}\;\mathrm{convenient}\;\mathrm{sampling}\;\mathrm{as}\;\mathrm{the}\;\mathrm{study}\;\mathrm{was}\\ & \;\mathrm{time}\;\mathrm{bound}. \end{array}$$

### 2.4 Saliva sample collection, preparation and storage

**Figure 1:**
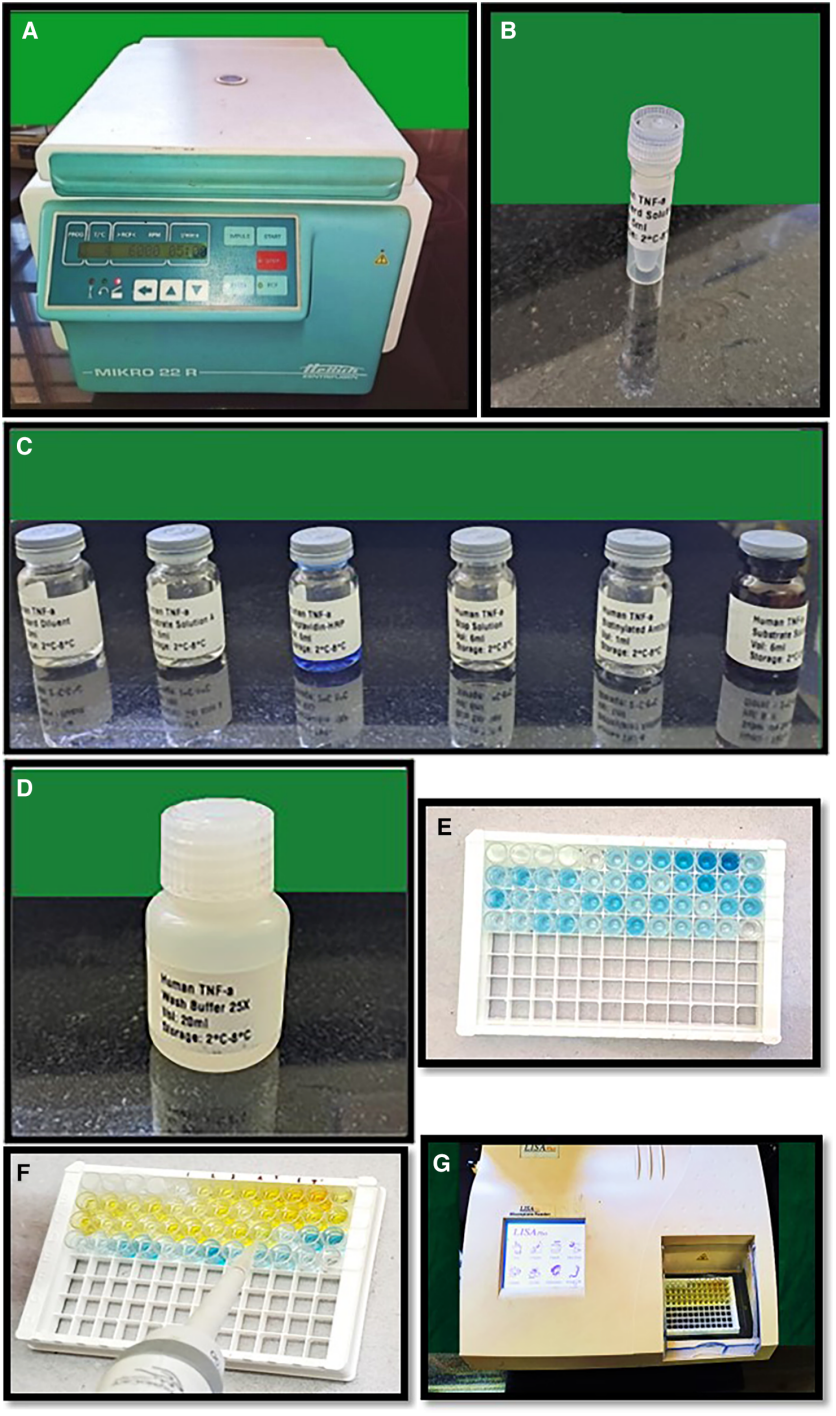
(**A**) Cooling centrifuge used for centrifugation of saliva samples, (**B**–**D**) reagents used for performing ELISA test, (**E**) showing addition of reagents (**A**,**B**) to the wells, (**F**) showing addition of stop solution with color change from blue to yellow in sample wells, (**G**)**-**LISAPlus microplate reader used for calculating the TNF-α.

Unstimulated saliva was collected from participants between 10:00 AM and 12:00 PM. The sample was collected by a single investigator from the participants to reduce the variation among investigators. Prior to collection, participants were instructed to rinse their mouths with water. The patients were then instructed to hold their saliva in their mouths for a few minutes before spitting it into sterile disposable centrifuge tubes. The saliva samples were collected over a period of 15 months beginning from 18.1.21 to 12.04.22. Approximately 3 ml of the collected saliva was subsequently centrifuged in a cooling centrifuge (MIKRO 22R Hettich Zentrifugen) at 6,000 rpm for 10 min at 4°C (Figure 1A). To analyse TNF-α levels using an ELISA kit, the supernatant generated from the centrifugation was pipetted and stored in 2 ml aliquots in a −80°C freezer. Each sample underwent one freeze‒thaw cycle, and the assay was conducted at room temperature (18).

### 2.5 TNF-α estimation

The estimation of salivary TNF-α was conducted using the Human Tumor Necrosis Factor Alpha ELISA Kit provided by Biocodon Technologies (BC-EH103522) which included the reagents used- Biotin labelled anti TNF-α antibody, Standard Diluent, washing buffer, standard solution, streptavidin HRP reagent, chromogenic reagent A, chromogenic reagent B and stop solution. This kit is specifically designed to quantify human TNF in saliva. The ELISA kits were precoated with capture primary antibody and the human TNF-α monoclonal antibody. To perform the assay, standards (Figure 1B) were added to the wells that had been coated with the antibodies. Subsequently, the samples were added, followed by the addition of biotin-labelled TNF-α antibody and streptavidin HRP (horseradish peroxidase) (Figure 1C). The mixture was then allowed to incubate to allow the immunological complex to develop. After incubation, the solution was discarded, and the plate was washed with wash buffer five times to remove any free enzyme (Figure 1D). The addition of reagents A and B to the wells caused the solution to turn blue (Figure 1E), and the addition of the stop solution turned it yellow (Figure 1F). Notably, there was a positive correlation between the human TNF-α concentration and the color of the solution. The optical density (OD) values were calibrated in the ELISA microplate reader and the OD values were converted into concentration of TNF-α.

The TNF-α concentration of the samples was determined based on the OD values of the samples as determined by the microplate reader (LISA Plus Microplate reader) (Figure 1G).

### 2.6 Statistical analysis

The statistical analysis was performed with SPSS version 20.0 (IBM Chicago). Descriptive statistics of the TNF-α levels in individual groups (control group, OSMF group and SCC group) are presented as the mean, median and standard deviation. Independent *t*-tests were used for the comparison of TNF-α levels among different grades and stages of OSMF patients. As the TNF-α levels were having higher standard deviations indicating skewed distribution Kruskal–Wallis test was done to evaluate the association between the control, OSMF and OSCC cases. One way ANOVA and Posthoc Tukey HSD test was used to compare the different grades of OSCC for the levels of TNF-α. *P* values <0.05 were considered statistically significant.

## 3 Results

The study included a cohort of 45 patients who were included based on the criteria mentioned above—15 individuals diagnosed with OSMF, 15 with SCC of the oral cavity and oropharynx, and 15 healthy controls. Among the participants, there were 31 males and 14 females, ranging in age from 20 to 64 years. The mean age of onset for OSMF was 42.47 ± 13.31 years, while for squamous cell carcinoma, it was 54.41 ± 7.23 years. Of the 15 individuals in the OSMF and SCC groups, 13 were males and 2 were females, indicating a clear male predominance. Notably, all patients diagnosed with OSMF and SCC were habitual users of smokeless tobacco and/or betel nuts, with the exception of one OSMF patient.

**Figure 2:**
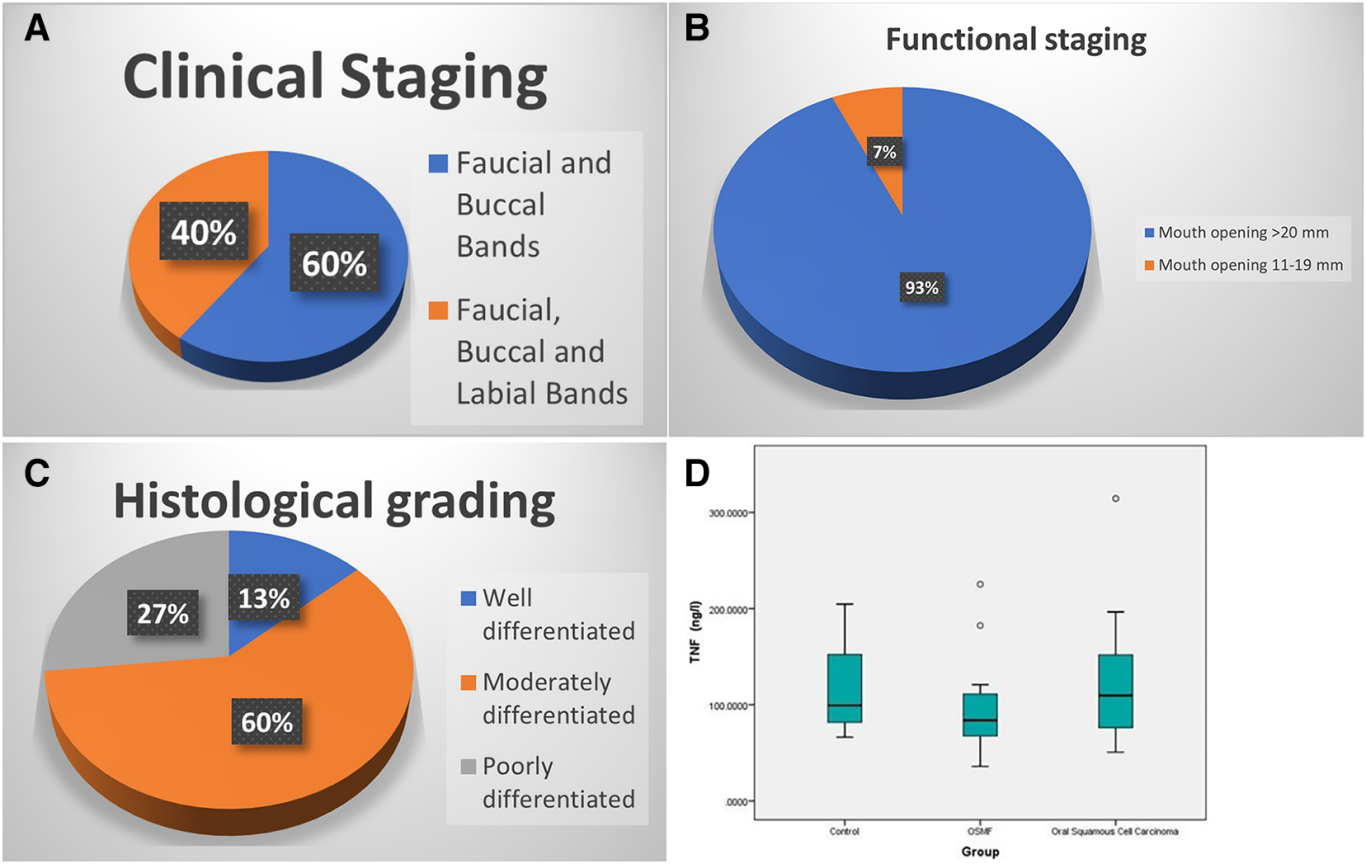
(**A**,**B**) Pie charts showing distribution of involvement in OSMF group by clinical staging and functional staging, (**C**) pie chart showing distribution of histological grading among SCC group, (**D**) box plot showing the TNF-α values between controls, OSMF and SCC. One outlier value of 2,807 has been eliminated from control group to make the graph better.

The clinical staging of the 15 patients with oral submucosal fibrosis (OSMF) revealed that 9 of them exhibited faucial and buccal bands, placing them in Stage 2. On the other hand, 6 patients displayed buccal, faucial, and labial bands, categorizing them in Stage 3 (Table 1, refer to Figure 2A).

**Table 1:** Comparison between the salivary TNF-α concentration and different stages of OSMF.

	** **		*N*	Mean ± SD	Median (IQR)	Range (min–max)	*T* value	*P* value
Clinical Staging	Stage 1	Faucial bands	0				−1.917	0.027
	Stage 2	Faucial and buccal bands	9	70.4 ± 20.7	69.2 (61.1, 77.1)	35.9–111.7		
	Stage 3	Faucial, buccal and labial bands	6	137.87 ± 54.06	115.75 (101.2, 182.4)	86.8–225.3		
Functional staging	Stage A	Mouth opening >20 mm	14	98.15 ± 51.37	80.55 (66, 111.7)	35.9–225.3	0.213	0.834
	Stage B	Mouth opening 11–19 mm	1	86.8				
	Stage C	Mouth opening <11 mm	0					

*P* value <0.05 was considered significant. Independent *T*-test.

Furthermore, functional staging, which was determined based on mouth opening, indicated that 14 out of the 15 patients (93.3%) had a mouth opening exceeding 20 mm, classified as Stage A. Only one patient fell into Stage B, with a mouth opening ranging from 11 to 19 mm (Table 1, refer to Figure 2B).

The SCC patients were classified according to Broder's Classification, based on their histological features. Among the 15 SCC patients, 9 had moderately differentiated carcinoma, 4 had poorly differentiated carcinoma, and 2 had well-differentiated SCC (Figure 2C). The tongue was the most common site of occurrence, followed by the buccal mucosa and oropharynx.

### 3.1 Comparison of the salivary TNF-α concentration with different stages of OSMF

Independent *t*-tests were also conducted to compare the TNF-α levels among patients with OSMF. The results revealed no significant difference among the functional staging groups. However, a significant difference was observed among the clinical staging groups (test value of −1.917 and *p* value of 0.027). On the other hand, the difference in TNF-α levels between the functional staging groups, which included patients in Stage A and Stage B, was not significant, with a test value of 0.213 and a *p* value of 0.834. (Table 1)

### 3.2 Comparison between salivary TNF-α levels and various histologic grades of SCC in the oral cavity and oropharynx

A comparison was conducted on the TNF-α (ng/L) concentration among three subgroups within the SCC group: mild, moderate, and poorly differentiated. The results revealed no significant difference between these subgroups, with a test value of 0.77 and a *p* value of 0.485. However, it is worth noting that the highest mean values were observed in the poorly differentiated squamous cell carcinoma subgroup (158.8 ± 103.98), followed by the moderately differentiated (111.84 ± 56.47) and well differentiated (97.85 ± 24.4) subgroups (Table 2). The One way ANOVA and *post hoc* Tukey HSD tests used to examine the differences between subgroups revealed that the most substantial difference was observed between the well-differentiated and poorly differentiated groups (60.95), although this difference was not found to be statistically significant. Additionally, the comparisons between the Moderately differentiated and Poorly differentiated groups (46.956, not significant) and between the Well-differentiated and Moderately differentiated groups (13.994, not significant) also showed no statistically significant differences.

**Table 2:** Comparison of the levels of salivary TNF-α within various histologic grades of SCC of the oral cavity and oropharynx.

	Well differentiated (*N* = 2) Mean ± SD	Moderately differentiated (*N* = 9) Mean ± SD	Poorly differentiated (*N* = 4) Mean ± SD	F/Welch statistics (*represents welch test)	*P* value	Well differentiated vs. moderately differentiated difference (*p* value)	Well differentiated vs. poorly differentiated difference (*p* value)	Moderately differentiated vs. poorly differentiated difference (*p* value)
TNF (ng/L)	97.85 ± 24.4	111.84 ± 56.47	158.8 ± 103.98	0.77	0.485	−13.99 (0.965)	−60.95 (0.586)	−46.96 (0.521)

*P* value <0.05 was considered significant. One—way ANOVA and Tukey test.

### 3.3 Comparison of TNF-α levels between the OSMF, SCC and control groups

The Kruskal‒Wallis test was used to compare TNF-α (ng/L) among the three groups: the OSMF, SCC, and control groups. The results indicated no significant difference (chi-square value of 2.422 and *p* value of 0.298). Interestingly, the SCC group exhibited the highest median values (109.6 with IQR of 74.4–168.8), followed by the control group having median of 102.7 (with an IQR of 81.7–162.7)groups (Table 3, refer to Figure 2D).

**Table 3:** Comparison of the levels of salivary TNF-α between the OSMF, SCC and the control groups (Kruskal–Wallis test).

		*N*	Mean ± SD	Median (IQR)	Range (min-max)	Chi square	*P* value
TNF (ng/L)	Control	15	294.69 ± 695.09	102.7 (81.7, 162.7)	66.3–2,802.4	2.422	0.298
	OSMF	15	97.39 ± 49.59	84 (66, 111.7)	35.9–225.3		
	Oral squamous cell carcinoma	15	122.5 ± 68.69	109.6 (74.4, 168.8)	50.8–314.4		

*P* value <0.05 was considered significant. Kruskal–Wallis test.

## 4 Discussion

Despite extensive research conducted over many years, particularly in South Asia, oral submucosal fibrosis (OSMF) has not been studied on a global scale. Although the incidence of this condition is on the rise, there has been little progress in effectively managing it (19). A recent 2021 study conducted by Rai et al. investigated the incidence of OSMF among boys aged 9–12 years attending school in a rural village. Notably, all of these boys reported a history of chewing tobacco and betel nut in a group setting (19). Furthermore, in 2019, Talla et al. in 2020 and Kariya et al. documented cases of OSMF in children as young as 5 years old. However, the oldest reported case of OSMF in a pediatric patient dates back to 1985, involving a 4-year-old girl, as reported by Hayes et al. (20).

Several studies have been conducted to determine the average age at which oral submucosal fibrosis (OSMF) occurs. Saalim et al. (21), Angadi et al. (22), and Pindborg et al. (23) investigated this topic. Saalim et al. (21) reported a mean age of occurrence of 35.51 ± 11.26 years (21), while Angadi et al. reported a mean age between 35 and 54 years (22). Pindborg et al. reported a wide range of 14–78 years, with a mean age of 46 years (23). Our study corroborates these previous findings, highlighting the average age of occurrence as 42.47 ± 13.31 years. This aligns with the notion that OSMF tends to manifest during middle adulthood, although it is important to note that cases can still arise earlier or later in life. By consolidating the results of these studies, we gained a comprehensive understanding of the age demographics associated with OSMF. The male-to-female ratio was found to be greater in studies conducted by Ranganathan et al. (24) and Tariq et al. (25). Similarly, our present study revealed that out of the 15 participants, 13 were males, while only 2 were females.

In a study conducted by Saalim et al. (21), the majority of patients (189 out of 300) were found to use a substance called “gutka”, which consists of areca nut, tobacco, catechu, and slaked lime. The remaining patients were using “supari” (areca nut), “mawa” (tobacco, slaked lime, and areca nut), or betel quid. Interestingly, our own study revealed that all patients diagnosed with OSMF (except one) were using either areca nut or betel quid. Furthermore, a significant number of patients (8 out of 15) reported a history of betel quid use, with some individuals using it for as long as 30 years. On the other hand, 6 out of the 15 patients reported using only areca nuts. These findings highlight the prevalence of areca nut and betel quid use among patients with OSMF.

Due to the widespread habit of chewing betel nuts and tobacco, oral squamous cell carcinoma (OSCC) is the most common form of cancer in the Southeast Asian and Pacific regions (26). A study conducted by Cancela et al. using population-based cancer registries revealed that patients from developing countries are diagnosed with cancer at an earlier stage than patients from developed countries are (27). Additionally, the incidence of OSCC among young men has been increasing, particularly in western India and the northern region (28). In a study conducted by Arduino et al. in 2008, the average age of diagnosis for patients with OSCC was 66.90 ± 11.72 years (29). However, in North America, OSCC typically presents in individuals in their 7th and 8th decades of life (30). Similarly, our study revealed that the average age at which OSCC occurred was 54.4 ± 7.23 years. OSCC has a male predilection (12). In 2012, Byakodi et al. reported that the male: female ratio was 2.6:1 (31). Our study also revealed a male predominance, as 13 of the patients who had OSCC were males and 2 of the patients were females.

In a study conducted by Gupta et al. in India, a clear correlation was discovered between the consumption of tobacco and oral squamous cell carcinoma (OSCC). This correlation was observed in terms of chewing frequency, duration, and age at first use. The study revealed that individuals who smoked ≥10 cigarettes or smoked bidis per day for more than 25 years had a significantly greater risk of developing oral cancer (8). In a study conducted by Gupta et al. in India, a clear correlation was detected between the consumption of tobacco and oral squamous cell carcinoma (OSCC). This correlation was observed in terms of chewing frequency, duration, and age at first use. The study revealed that individuals who smoked ≥10 cigarettes or smoked bidis per day for more than 25 years had a significantly greater risk of developing oral cancer (8). Our own research further supports these findings. We found that the maximum frequency of tobacco use, both in smoke and smokeless forms, was approximately 10–15 times per day. Among the patients we studied, the average frequency of tobacco use was 6.66 ± 5.25 times per day. Additionally, the mean duration of tobacco consumption, whether smoke or smokeless, was 16.43 ± 11.14 years. These results highlight the alarming prevalence of tobacco use and its detrimental effects on oral health. It is crucial for individuals to be aware of the risks associated with tobacco consumption and to take necessary steps to prevent oral cancer.

A study conducted in 2015 by Pratik et al. in Jaipur, Rajasthan, revealed some concerning findings. It was found that both smoking and smokeless tobacco were used by 51.4% of the individuals, regardless of sex. Furthermore, oral mucosal lesions were prevalent in 9.9% of the population (32). In our own study, we observed that all patients diagnosed with oral squamous cell carcinoma (OSCC) had a history of tobacco consumption, either in the form of smoking or smokeless tobacco (SLT). Interestingly, SLT was more commonly used, with all patients reporting its use. This included the consumption of plain tobacco or commercially available products such as gutkha. Moreover, the majority of male patients and all female patients consumed both betel nut and slaked lime in combination. This combination is known to be particularly harmful to oral health. Additionally, male patients had a greater tendency to consume alcohol on a regular basis. These findings highlight the alarming prevalence of tobacco use and its association with oral health issues, specifically OSCC. It is crucial to address these habits and raise awareness about the risks they pose.

The synthesis and breakdown of extracellular matrix proteins interact intricately to promote the development of OSMF. Due to an imbalance between increased production and deposition and reduced breakdown of the components of the extracellular matrix, fibrosis results in the buildup of collagen (33). A balance of the mediators of inflammation likely plays a critical role in controlling the onset and progression of fibrosis under any fibrotic condition. Cytokines play a significant role in modulating fibroblast activity, such as in the proliferation and migration of fibroblasts and matrix formation (34). TNF-α and IL-1 have been reported to increase fibroblast growth in culture (35). Additionally, in 2020, Piget et al. demonstrated via *in vivo* investigation that subcutaneous injection of TNF-α caused local fibroblast, capillary, and epidermal cell proliferation as well as a large increase in hydroxyproline levels (36). In 1996, Chou et al. reported that TNF-α inhibits collagen adhesion and phagocytosis, and they hypothesized that this inhibition of the collagen phagocytic pathway may be a factor in the development of fibrosis (37).

Saliva offers several advantages over plasma as a diagnostic fluid due to its cost-effectiveness and noninvasive collection method. The effectiveness of saliva as a diagnostic tool can be attributed to its constant presence in close proximity to oral neoplasms and premalignant conditions. Therefore, the utilization of diagnostic biomarkers could greatly aid in identifying OSMF and early OSCC, especially when there are no apparent physical symptoms of the disease at this stage (38).

A study conducted by Sodhi et al. in 2012 revealed that patients with oral submucous fibrosis (OSMF) exhibited higher levels of TNF-α than did control participants (33). However, in 2012, Brailo et al. reported no difference in salivary TNF-α levels between patients with leukoplakia and those with oral cancer. Interestingly, TNF-α levels in the plasma of individuals in the control group were found to be greater than those in patients with oral cancer (39).

In line with these findings, our study aimed to compare salivary TNF-α levels among three groups: the OSMF group, the oral squamous cell carcinoma (OSCC) group, and the control group. Surprisingly, our results showed no significant difference in salivary TNF-α levels among these groups (chi square value of 2.422 and *p* value of 0.298).

In a 2012 study conducted by Swastika et al., the expression of TNF-α was correlated with the grade of oral submucous fibrosis (OSSF). Higher expression of TNF-α was observed in Grade IV OSMF patients according to the Khanna and Andrade classification than in Grade 2 OSMF patients (40). Similarly, in 2015, Kaur et al. demonstrated that the levels of TNF-α, IL-6, and IL-8 in saliva and serum were elevated in patients with OSMF, oral lichen planus, and oral leukoplakia compared to those in the control group (14).

Furthermore, in 2019, Deepthi et al. discovered that, compared with those in the control group, the levels of TNF-α in the OSCC (oral squamous cell carcinoma) and leukoplakia groups were elevated with increasing histologic grade (17). Concurrently, our own study also revealed the OSCC group exhibited the highest median values of salivary TNF-α, which was however, followed by the control group. Among the OSMF group, an increase in TNF-α levels in patients exhibiting stage 3 (clinical stage: faucial, buccal, and labial bands) disease according to the Open Signatures Scale (OSTF) compared to those exhibiting stage 2 (clinical stage: faucial and buccal bands) was seen.

To better characterize the role of TNF-α in OSMF and SCC, additional investigations with larger sample sizes are needed since this study included only a small number of patients. Overall, these studies provide valuable insights into the relationship between TNF-α expression and various oral conditions, highlighting the need for further research in this area.

Numerous studies have also evaluated the use of a number of TNF-α inhibitors in OSMF therapy (29). In the future, dentists, biotechnology companies and pharmaceutical firms may collaborate with academic laboratories to successfully translate these fascinating insights into the clinic (38).

## 5 Conclusion

Our study attempted to correlate salivary TNF-α levels with different stages of OSMF and SCC of different histologic grades in the oral cavity and oropharyngeal region. The findings indicate that TNF-α levels increase with increasing clinical stage of OSMF. However, there were no significant differences observed among the OSMF, SCC (squamous cell carcinoma), or control groups.

## Data Availability

The raw data supporting the conclusions of this article will be made available by the authors, without undue reservation.
